# Green Fluorescent Protein-Based Viability Assay in a Multiparametric Configuration

**DOI:** 10.3390/molecules23071575

**Published:** 2018-06-28

**Authors:** Rita Csepregi, Viktória Temesfői, Miklós Poór, Zsuzsanna Faust, Tamás Kőszegi

**Affiliations:** 1 Department of Laboratory Medicine, Medical School, University of Pécs, Ifjúság u. 13, H-7624 Pécs, Hungary; ritacsepregi93@gmail.com (R.C.); vtemesfoi@gmail.com (V.T.); faust.zsuzsanna@pte.hu (Z.F.); 2 János Szentágothai Research Center, University of Pécs, Ifjúság u. 20, H-7624 Pécs, Hungary; poor.miklos@pte.hu; 3 Department of Pharmacology, Faculty of Pharmacy, University of Pécs, Szigeti út 12, H-7624 Pécs, Hungary

**Keywords:** microplate assay, flow cytometry, green fluorescent protein, cell viability

## Abstract

Green fluorescent protein (GFP) is considered to be suitable for cell viability testing. In our study, GFP transfected A549 lung carcinoma cell line was treated with sodium fluoride (NaF), cycloheximide (CHX) and ochratoxin A (OTA). GFP fluorescence, intracellular ATP, nucleic acid and protein contents were quantified by a luminescence microplate assay developed in our laboratory. Flow cytometry was used to confirm the findings and to assess the intensity of GFP during different types of cell death. A 24 h NaF and CHX exposure caused a dramatic decrease in ATP contents (*p* < 0.05) compared with those of the controls. GFP fluorescence of the cells was in close correlation with total protein; however, GFP/ATP increased at NaF and decreased at CHX treatments (*p* < 0.05). ATP/protein and ATP/propidium iodide (PI) were largely decreased at NaF exposure in a dose-dependent manner (*p* < 0.05), while CHX and OTA showed markedly fewer effects. Both treatments caused apoptosis/necrosis at different rates. NaF induced mainly late apoptosis while OTA, mainly apoptosis. CHX effects varied by the incubation time with 100-fold elevation in late apoptotic cells at 24 h treatment. GFP intensity did not show a significant difference between live and apoptotic populations. Our results suggest when using GFP, a multiparametric assay is necessary for more precise interpretation of cell viability.

## 1. Introduction

Cell viability and cytotoxicity tests are frequently used assays, based on the general considerations about cell death. Live and dead cells can be distinguished by certain dyes that require intracellular enzymatic activity or by their characteristics to enter the cells only if the plasma membrane integrity is compromised. Cell adherence and the capability to form colonies also gives information on viability. Cytotoxicity can also be assessed by detecting the intracellular protein release. Another significant goal in this regard is the application of methods including metabolic parameters as well [1].

The molecular basis of green fluorescent protein (GFP) tag/protein expression provides us with a picture about the background of how GFP fluorescence can be used in connection with cell viability [2,3,4,5,6,7]. Depending on the construct used for transfection, it can be co-expressed with a specific protein, regulated by the promoter of the protein of interest or randomly integrated into the genome with an own promoter sequence. In our constellation, the HIV-derived lentivectors are stably transfected into A549 lung carcinoma cell line under the control of a cytomegalovirus (CMV) promoter [8]. As Soboleski and colleagues demonstrated, the yield of GFP messenger ribonucleic acid (mRNA) and the average fluorescence intensity of GFP protein were proportional in in vitro studies, where GFP expression was put under the control of three different eukaryotic promoters. Thus, GFP fluorescence intensity was shown to be in correlation with transcriptional activity. Based on these findings, it is a powerful and easily measurable quantitative reporter to detect changes in gene expression [9]. Transcriptional activity and protein synthesis depend strongly on the general energy supply of the cells. Energy is stored in the chemical bonds of adenosine triphosphate (ATP) molecules, which are end products of metabolic pathways. Intracellular ATP level is widely used to determine the viability and to estimate the metabolic efficiency of cells [10,11,12,13]. The luminescence-based ATP assays are the most precise and sensitive assays in the microplate technology available in cell viability studies, although, detecting ATP as a marker of viability has its limitations. It has to be emphasized, that depletion of the ATP level does not always infer lethal alterations [1]. In our study, using metabolic and protein synthesis inhibitors and a generally toxic compound we analyzed the cellular metabolism and protein synthesis pathways from the endpoint to obtain information on the changes of GFP expression/intensity by affecting the production of ATP. Thus, including other cytotoxicity/viability markers, we can infer the applicability of GFP fluorescence regarding cell viability.

Anti-metabolic effects of sodium fluoride (NaF) manifests mainly through influencing the activity of the enzymatic system [14]. This involves the inhibition of glycolytic enzymes which largely contribute to the ATP production, especially in tumorigenic cells, where most of the energy yield depends on glycolysis, even when the oxygen supply is sufficient, and oxidative phosphorylation is functioning (Warburg effect) [15,16]. Cycloheximide (CHX) is a widely used compound to inhibit eukaryote protein synthesis. It is known to stop translation during the elongation phase by binding the 60S ribosomal subunit [17]. The complex effect of ochratoxin A (OTA) is mediated particularly through the induction of oxidative stress. Several processes are involved in its toxicity, such as lipid peroxidation, inhibition of protein synthesis, mitochondrial pathways, and damage of the DNA [18,19].

Apoptosis and necrosis can be differentiated by well-characterized physiological and biochemical processes. During necrosis, the main features are disruption of the plasma membrane and intracellular organelles, loss of cellular content or swelling can be observed. 

To diagnose apoptosis, there are some good points to rely on, such as the morphological changes, caspase activity, and phosphatidyl serine (PS) externalization. Apoptotic cells keep their intact plasma membrane until the late phases of the process. Before dying cells meet the criteria to be considered dead, events may be reversible in some cases. Based on the recommendations of the Nomenclature Committee on Cell Death, cells can be determined as dead among others by the loss of the plasma membrane integrity which can be identified with viability dyes, such as propidium iodide (PI) and 7-aminoactinomycin D (7AAD) [20].

In our previous study, calcein acetoxymethyl esther (CAM) dye was used for viability testing, but its applicability to discriminate live/dead cells seemed to be questionable. The ATP depleted Madin-Darby canine kidney (MDCK), and liver hepatocellular carcinoma (HepG2) cells took up the CAM dye at the same kinetics as the untreated control cells, and the same extent of fluorescence was detected in these two groups [10].

In the present work, we attempted to find coherence between the intensity of GFP fluorescence, intracellular ATP yield, nucleic acid content (approximate cell number), and total protein level for estimation of the viability of cells after treatment with metabolic poisons. To answer the question, we modified and combined our previously published plate reader-based cell viability assay [10,11] with GFP fluorescence detection. To specify our findings further, we invoked flow cytometry as well which allowed us to study the GFP signal during the toxin-induced apoptotic and necrotic processes.

## 2. Results

### 2.1. Microplate Assay

The ATP content showed significant changes in each treatment. The strongest depletion was detected during NaF exposure. At 20 mM NaF concentration, ATP was reduced to approximately 2% compared with the control. CHX in 4 h elevated the ATP level, while 24 h incubation caused a significant decrease. Treatments with OTA reduced the intracellular ATP levels as well. Each compound caused dose-dependent alterations, as shown in Table 1.

**Figure 1:**
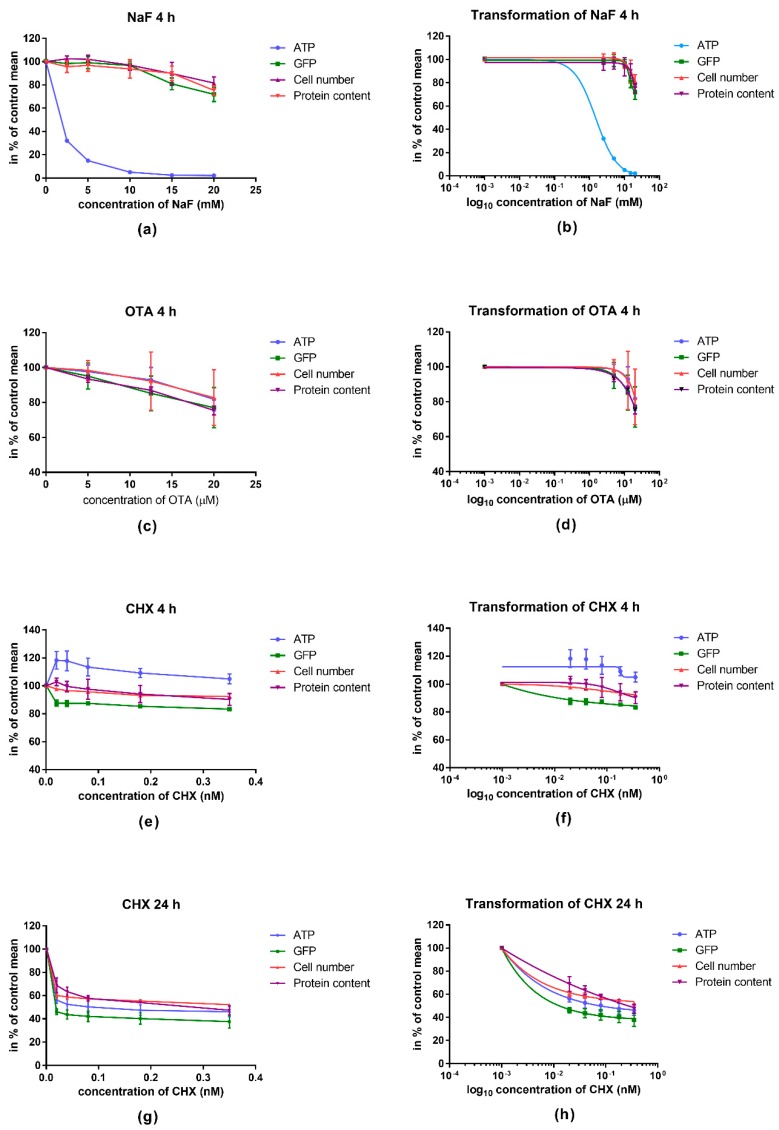
Dose-response fitting of the results regarding the four measured parameters. (**a**) Graphs of absolute parameters of sodium fluoride (NaF); (**c**) Ochratoxin A (OTA); (**e**) Cycloheximide (CHX) 4 h; (**g**) CHX 24 h. Dose-response curves of (**b**) NaF; (**d**) OTA; (**f**) CHX 4 h; (**h**) CHX 24 h, created by log10 transformation and nonlinear curve fitting. Correlation coefficients (R^2^) in NaF treatment: Adenosine triphosphate (ATP) 0.9993, green fluorescent protein (GFP) 0.8925, cell number 0.7259, protein content 0.7257. Correlation coefficients (R^2^) in OTA treatment: ATP 0.7004, GFP 0.5938, cell number 0.3022, protein content 0.9586. Correlation coefficients (R^2^) in CHX 4 h treatment; ATP 0.1225, GFP 0.9366, cell number 0.8959, protein content 0.4892. Correlation coefficients (R^2^) in CHX 24 h treatment; ATP 0.9666, GFP 0.9746, cell number 0.9898, protein content 0.9647. Data are represented in percentage of control mean ± SD of 4 independent experiments, *n* = 4 × 16 replicates for each concentration. Analysis was carried out by comparing the logIC50 values of the measured parameters using extra sum-of-squares *F* test (comparison of fits) (*p* < 0.0001).

We found a strong correlation between the GFP fluorescence intensity, cell number, and total protein content during treatments and a remarkably significant difference could be detected in the case of NaF exposure influencing ATP levels correlated to GFP intensity, cell number and protein content and also, at 4 h CHX treatment influencing ATP levels correlated to GFP intensity (*p* < 0.0001). Results are demonstrated in Figure 1.

**Figure 2:**
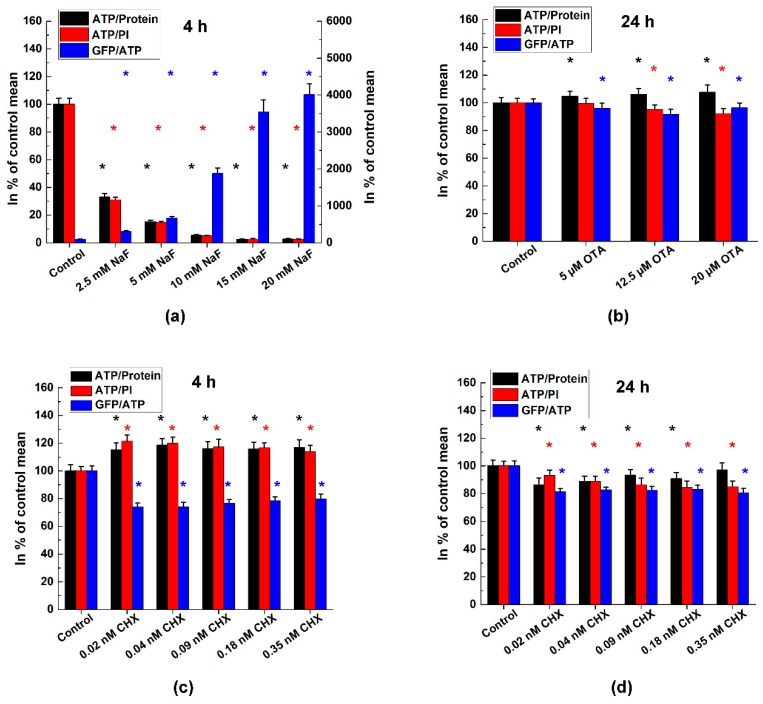
ATP/Protein, ATP/propidium iodide (PI) and GFP/ATP ratios in % of the control. We used Hanks’ solution as control of the NaF treatment. Mean ± SD of 4 independent experiments, *n* = 4 × 16 replicates for each concentration. Effects of (**a**) NaF; (**b**) OTA; and (**c**,**d**) CHX treatments of A549-GFP cell line. *: significant change vs. control (one-way ANOVA test, *p* < 0.05).

Figure 2 represents the ratios of the measured parameters. Regarding NaF treatment, we observed a strong decrease in the ATP/protein and ATP/cell number ratios while the GFP/ATP quotient showed significant elevation, interestingly. OTA increased the ATP/protein ratio significantly, other quotients showed a decrease, but the change is less steep compared to the alterations caused by NaF. Short-time incubation with CHX caused elevated ATP/protein and ATP/cell number ratios but reduced the GFP/ATP quotient significantly. During 24 h CHX treatment, along with the lower ATP content, the corresponding ratios showed to be lower as well.

### 2.2. Flow Cytometry

**Figure 3:**
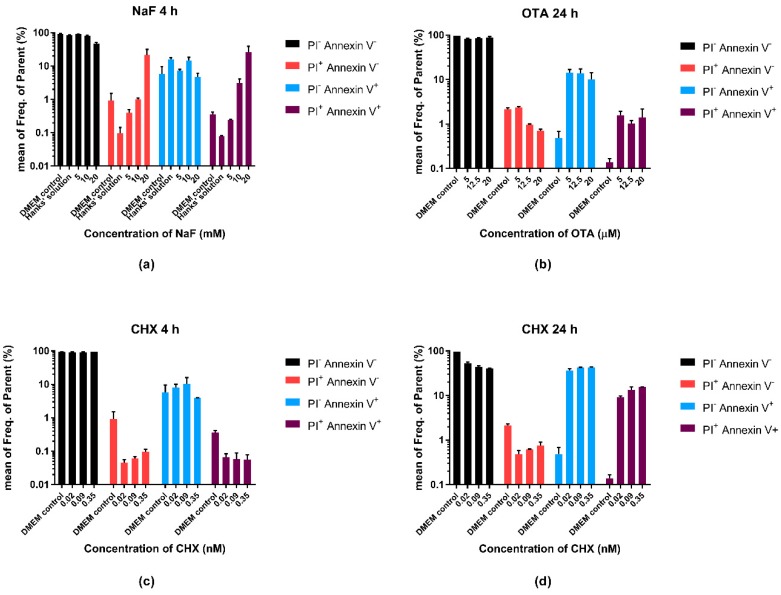
Effects of different treatments on cell death; (**a**) NaF; (**b**) OTA; and (**c**,**d**)CHX. Percentage distribution of Annexin V^+^, PI^+^, negative and double positive cells in the parental gate (A549 cells defined by side scatter area (SSC-A)/forward scatter area (FSC-A) presented on a log10 scale. Dulbecco’s Modified Eagle Medium (DMEM) is the absolute control of the measurements, Hanks’ solution is the control of the NaF treatment.

In the flow cytometry experiments, first, we investigated the effect of the compounds on cell death regarding four different populations, defined by fluorescence minus one (FMO) controls. These populations are the PI^−^ Annexin V^−^ (live), PI^+^ Annexin V^−^ (cells with compromised membrane, without PS externalization), PI^−^ Annexin V^+^ (cells with externalized PS) and the PI^+^ Annexin V^+^ (double positive) cells. Figure 3 indicates the percentage distribution of these populations in the treated cells. An example of the gating strategy can be seen in Figure S1.

NaF showed apoptosis-inducing and necrotic effects, as it is seen by the dose-dependent increase of the necrotic and late apoptotic populations, while the percentage of the apoptotic cells remained approximately constant across different concentrations. OTA seemed to induce apoptosis rather than necrosis in this experimental design and circumstances, as we found the necrotic population to decrease dose-dependently, while the apoptotic and the double positive cell number tended to increase compared with the control. The effect of CHX was time-dependent. Comparing the short (4 h) and the long (24 h) treatments, an approximately tenfold growth of the necrotic and a hundredfold growth of the late apoptotic population is remarkable with an elevation in the number of the apoptotic cells as well.

**Figure 4:**
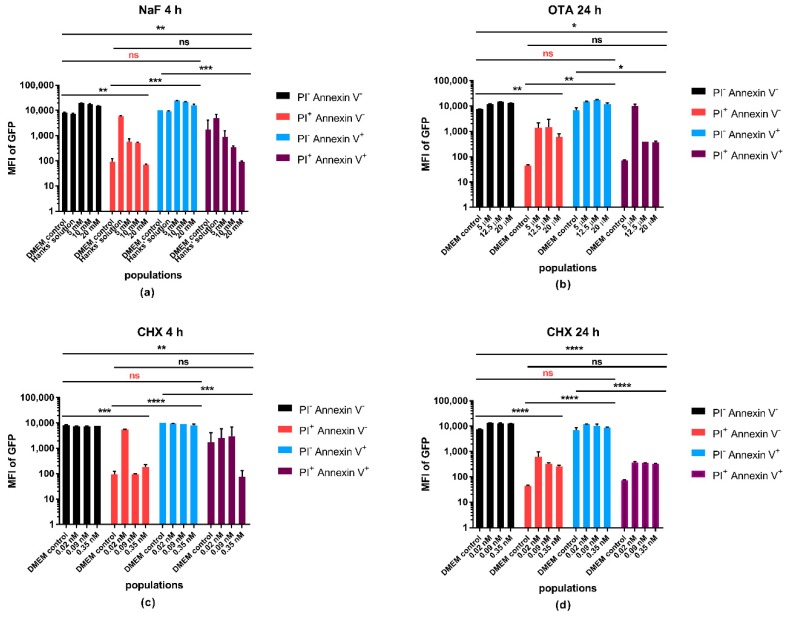
Fluorescence intensity of GFP within the PI^−^ Annexin V^−^, PI^+^ Annexin V^−^, PI^−^ Annexin V^+^ and PI^+^ Annexin V^+^ double-positive populations. Effects of (**a**) NaF; (**b**) OTA; and (**c**,**d**) CHX on the fluorescence intensity of GFP in A549 cell line. DMEM serves as absolute control of the measurements, Hanks’ solution as the control of the NaF treatment. Fluorescence data showed nonparametric distribution, thus, means of medians are shown on the graph (two technical replicates for each treatment). Error bars represent the standard deviation (SD). One-way ANOVA and Tukey’s post hoc test were performed to obtain between-group differences. *: significant difference (*p* < 0.05), **: significant difference (*p* < 0.01), ***: significant difference (*p* < 0.001), ****: significant difference (*p* < 0.0001). ns: no significance, ns: no significant difference between live and apoptotic populations.

We investigated the fluorescence intensity of expressed GFP in the different populations defined by PI and Annexin V positivity, as indicated in Figure 4. In all the treatments, a significant difference was observed between the negative vs. double positive, the negative vs. PI^+^ Annexin V^−^, the PI^+^ Annexin V^−^ vs. PI^−^ Annexin V^+^ and the PI^−^ Annexin V^+^ vs. double positive populations. There was no significant difference detected between the PI^+^ Annexin V^−^ vs. double positive and the live vs. PI^−^ Annexin V^+^ apoptotic populations, suggesting that the loss of the GFP signal meets the criteria of cell death, where membrane damage occurs, while during the apoptotic process, the GFP signal still can be detected. Figure S2 shows an example of the GFP intensity represented by histograms in control samples and in different treatments.

## 3. Discussion

Microplate assays are widely used in drug and cytotoxicity testing. It is a fast screening method providing high-throughput data from small quantities of test samples. We developed a sensitive and rapid extraction method which allows us to explore the intracellular protein and ATP contents in one step to reduce time and to avoid the possibility of errors. In this study, we examined the impact of three different chemicals on GFP transfected A549 cell line to reveal the correlation between the GFP fluorescence and a metabolism-related viability parameter. We studied the GFP fluorescence intensity during apoptosis and necrosis using the commercial live/dead discrimination and apoptosis detection.

The manner of cell disruption is a cornerstone of microplate-based assays investigating intracellular biochemical parameters. Several cell lysis techniques exist depending on the purpose of the experiment or cell type: Mechanical, physical, chemical, and biological methods. There are experiments using mild procedures, such as freezing, osmosis, fast ultrasonic lysis at low temperature or several types of detergents, like sodium dodecyl sulphate (SDS) and Triton X-100 [21]. As the development of a previously published method [10], we applied a boric acid buffer at alkaline pH supplemented with Triton X-100 and ethylene-diamine-tetra-acetic acid (EDTA) to mobilize and stabilize total protein and ATP contents of adherent cell cultures at the same time. This helps to avoid repeated pipetting, causing uneven distribution or loss of cells from the wells.

There is no surprise that the GFP signal intensity moves along with cell number and protein content of the cells. Its expression is under the control of an external promoter, but the general capability of the cells to transcribe and translate gene sequences into proteins has a clear impact on the synthesis of the fluorescent protein as well. In the microplate-based experiments, the most remarkable changes can be observed when NaF, as a general inhibitor of the enzymatic system, is used. The GFP/ATP ratio can still be higher or elevate dose-dependently, even if the ATP content drops down significantly without any change in the total protein level. This observation supports our suggestion that ATP determination solely may not always be an adequate viability parameter. 4 h treatment with CHX caused elevation of the ATP content, which can be attributed to its apoptosis inducing effects through the inhibition of protein synthesis. Apoptotic processes require energy to guide through the downstream program seamlessly [22,23]. On the other hand, increase of ATP does not obligatorily mean an increased production but might be the result of less utilization. Protein synthesis is strongly energy dependent therefore, if CHX inhibits the protein synthesis pathways then it might result in accumulation of unused ATP in the cells. The results of the 24 h CHX treatment let us conclude that we see a rather advanced apoptotic outcome with an increasing PI and Annexin V double positive population and reduced ATP/protein, ATP/cell number ratios. OTA is included into the study to learn how complex cytotoxic effects influence the fluorescence intensity of GFP. It is difficult to determine the exact cellular targets of the toxin and this study was not meant to investigate it further; however, we can ascertain that the decrease of the fluorescence intensity represented the cell destruction.

The main reason for the loss of GFP signal at the endpoint of cell death is the leakage, loss, and degradation of the intracellular content resulted from the perforation of the membrane. Apoptosis and other types of cell death, during which cells remain intact until the late phases, require more attention in this regard. The GFP fluorescence showed much lower differences between the live and apoptotic populations than between live and necrotic, apoptotic and necrotic, or any other combinations. GFP fluorescence can be detected from these cells as long as the transcriptional and translational processes are functioning to satisfy the needs of the cells during the programmed process, no harsh disruption is happening, and cellular integrity is kept. In the flow cytometry investigation, it is possible to sharply demarcate apoptotic and necrotic cell groups using appropriate markers, but in microplate assays if viability experiments are based only on the GFP intensity, apoptotic cells can also contribute with their fluorescence to falsely assume the whole population to be live or more viable.

While measuring ATP, it has to be taken into account that apoptotic processes require a higher elevated energy level than measured in the control cells; it is a prerequisite of programmed cell death [23]. Thus detecting higher ATP content does not always mean higher cell viability. The situation is similar regarding the fluorescence intensity of expressed GFP. Every observation made in cell viability and cytotoxicity testing has to be based on well-established protocols and multiparametric measurements to have a precise view on the tested effects and to avoid inaccuracy and irreproducible results.

## 4. Materials and Methods

### 4.1. Cell Culture

GFP transfected A549 adherent lung cancer cell culture (ATCC CCL-185) was a kind gift from Krisztián Kvell. The A549 cell line was sorted with FACSAria II (Becton Dickinson and Company, Franklin Lakes, NJ, USA) instrument to get nearly 100% of GFP positivity. The transfection was carried out using HIV-1-derived lentivectors [8]. A549-GFP cells were cultured in Dulbecco’s Modified Eagle Medium (DMEM, Sigma, Saint Louis, Missouri) with 10% fetal bovine serum (FBS), penicillin (100 U/mL), and streptomycin (100 µg/mL) at 37 °C and 5% CO_2_ in a humidified incubator.

### 4.2. Chemicals and Treatments

Ochratoxin A (OTA), cycloheximide (CHX), propidium iodide (PI), fluorescamine (fluram) and Dulbecco’s Modified Eagle Medium (DMEM) were purchased from Sigma-Aldrich. Sodium fluoride (NaF) was from Acros Organics (part of Thermo Fischer Scientific, Waltham, Massachusetts). NaF was used dissolved in glucose containing homemade Hanks’ solution during the treatments. For cellular measurements, we used phosphate-buffered saline (PBS, pH 7.4) and ATP measurement buffer (0.1 M Tris/acetate, 2 mM EDTA, 10 mM MgSO_4_, pH 7.75), fetal bovine serum (FBS, Pan-Biotech, Aidenbach, Germany), Bioluminescent ATP Assay Kit CLSII (Roche, Basel, Switzerland), Triton X-100 (Roche), and bovine serum albumin (BSA, Biosera, Nuaille, France) were used as received. Annexin binding buffer (10 mM 4-(2-hydroxyethyl)-1-piperazineethanesulfonic acid (HEPES), 140 mM NaCl, 2.5 mM CaCl_2_, pH 7.4) was homemade, Annexin V-Pacific blue was from Life Technologies (Carlsbad, California). Treatments were carried out in the concentrations described in Table 2.

### 4.3. Microplate Assay

Following the treatment, cells were washed three times with calcium and magnesium-containing PBS to remove the debris. Cells were solubilized using 200 µL 0.1% Triton X-100 non-ionic detergent containing borate buffer (pH 9.2) supplemented with 10 mM EDTA and were placed on a shaker for 5 min. The microplate cytotoxicity assay is based on a multiparametric measurement [10]. ATP was determined by the luciferin/luciferase technique [10] adapted for microplate method. To measure the ATP content, lysed samples were transferred into 96-well white optiplates, 20 µL/well each. 200 µL of dissolved ATP reagent was added to the wells. The working concentration of PI staining was 2 µg/mL in PBS. The extracted samples were incubated for 5 min at room temperature in the dark, and after a short shaking they were measured on plate reader (Enspire Multimode reader, Perkin Elmer, (Waltham, Massachusetts) at λexc 530 nm and λem 620 nm wavelengths. Fluorescence intensity of GFP was measured at λexc 480 nm and λem 520 nm wavelengths. We used fluorescamine (0.3 mg/mL dissolved in acetone) to determine the intracellular protein content. Bovine serum albumin standard was used to generate linear regression calibration curve. Protein concentration was determined in the range of 20–100 mg/L. 20 µL/well borate buffer-lysed sample/standard was transferred into a 96-well standard plate. After pipetting 150 µL of 0.1% Triton X-100 containing borate buffer into the wells, 50 µL of fluram/acetone was rapidly added to each well. After a short shaking, fluorescence intensity was measured on the plate reader at λexc 385 nm and λem 490 nm wavelengths.

### 4.4. Flow Cytometry

Before the experimental procedure, depending on the treatment time 8 × 10^5^ and 6 × 10^5^ cells were pipetted into 6-well tissue culture plates (Sarstedt, Nümbrecht, Germany). Following overnight incubation, the medium was replaced with the treating medium, containing OTA, CHX, and NaF (as shown in Table 1).

Following incubation at 37 °C and 5% CO_2_, supernatant was collected separately from each well, cells were trypsinized, and centrifuged together with the previously obtained supernatant at 400 g and 4 °C for 5 min. The pellet was washed once in Annexin binding buffer (10 mM HEPES, 140 mM NaCl, 2.5 mM CaCl_2_, pH 7.4). Samples were analyzed immediately on a BD FACS Canto II flow cytometer (Becton Dickinson and Company). Analysis was carried out with FlowJo v10 data analysis software (FlowJo, LLC, Ashland, OR, USA).

To detect the externalized PS residues as apoptotic markers, the GFP expressing A549 cells were labeled with Pacific Blue conjugated Annexin V in Annexin binding buffer according to the manufacturers’ protocol, on ice. PI was used to specify the necrotic cells with compromised membrane at the working concentration of 1 µg/mL. During the spectral overlap compensation process A549 cells were treated with tumor necrosis factor related apoptosis inducing ligand (TRAIL) to serve positive control for apoptosis. Necrosis was generated by heat exposure for positive control of PI staining. FMO controls were applied to set the gates properly. The concentrations of OTA, CHX, and NaF for the flow cytometry measurements were selected based on the results of the microplate assay.

### 4.5. Statistical Evaluation

Treatments for microplate measurements were carried out on four plates and 16 technical replications. The obtained data were averaged separately and compared in percentages of the mean of the untreated control ± SD (100% ± SD). We used one-way ANOVA, where the control and the data of one type of treatment were compared. A paired T-test was performed for comparison of data sets for GFP, PI, and protein values with each other and the trend between GFP and PI, or GFP and total protein content in the cell line was examined. Flow cytometry experiments were performed in duplicates. To compare the distribution of different populations within treatments, percent distributions of technical replicates were averaged and the mean was presented on a log10 scale to make low values visible. Fluorescence intensity of GFP showed nonparametric distribution. Medians were averaged separately in each concentration and controls of each treatment. One-way ANOVA and Tukey’s post hoc tests were performed to obtain between-group differences. The level of significance was set at *p* < 0.05. The work was carried out in SPSS Statistics (version 22, IBM, Armonk, NY, USA) and GraphPad Prism 7 (version 7, GraphPad Software, San Diego, CA, USA) softwares.

## 5. Conclusions

There are some remarkable considerations to be mentioned. In the present study we learned the behaviour of intracellular ATP level in correlation with GFP expression as an indicator of viability. Our results suggest, that decrease in GFP fluorescence is not always in correlation with the intracellular ATP content. In the case of stable transfection of the fluorescent protein into the genome with an own promoter sequence, it can be used as a cell viability marker, but the impact of the tested compound on the type of cell death and the duration of treatment must be taken in account. Our suggestion is to perform multiparametric measurements when applying expressed GFP, along with conventional live/dead discrimination and including a metabolic parameter as well, to get more reliable information from the experiments.

## Figures and Tables

**Table 1:** Effects of various treatments on the adenosine triphosphate (ATP) content of A549-GFP cells. Data are expressed in % of the control. Mean ± SD of 4 independent experiments, *n* = 4 × 16 replicates for each concentration. ^a^: Significant change compared to the control (one-way ANOVA test, *p* < 0.05).

Treatment Groups			ATP Content (%)
NaF	4 h	Control	100.00 ± 3.03
		2.5 mM	31.94 ± 1.47 ^a^
		5 mM	14.94 ± 0.76 ^a^
		10 mM	5.06 ± 0.37 ^a^
		15 mM	2.44 ± 0.25 ^a^
		20 mM	2.11 ± 0.22 ^a^
CHX	4 h	Control	100.00 ± 3.39
		0.02 nM	118.32 ± 4.16 ^a^
		0.04 nM	117.87 ± 4.26 ^a^
		0.08 nM	113.46 ± 5.06 ^a^
		0.18 nM	109.25 ± 3.04 ^a^
		0.35 nM	104.95 ± 3.54 ^a^
CHX	24 h	Control	100.00 ± 2.53
		0.02 nM	56.18 ± 1.91 ^a^
		0.04 nM	52.56 ± 2.31 ^a^
		0.08 nM	50.37 ± 1.11 ^a^
		0.18 nM	47.46 ± 0.99 ^a^
		0.35 nM	48.30 ± 1.03 ^a^
OTA	24 h	Control	100.00 ± 1.99
		5 µM	97.65 ± 2.26 ^a^
		12.5 µM	92.99 ± 3.42 ^a^
		20 µM	81.98 ± 3.23 ^a^

**Table 2:** Concentrations and duration of treatments in the microplate assay and flow cytometry measurements.

Treatment	Time (h)	Microplate Assay	Flow Cytometry
OTA	24	5 µM, 12.5 µM, 20 µM	5 µM, 12.5 µM, 20 µM
CHX	24	0.02 nM, 0.04 nM, 0.09 nM, 0.18 nM, 0.35 nM	0.02 nM, 0.09 nM, 0.35 nM
	4		
NaF	4	2.5 mM, 5 mM, 10 mM, 15 mM, 20 mM	10 mM, 15 mM, 20 mM

## Supplementary Materials

The followings are available online, Figure S1: Gating strategy in the flow cytometry experiments. Flow stability was checked by forward scatter area (FSC-A)/Time gate, single cells were discriminated by FSC-Height/FSC-A, A549 cells were determined on side scatter area (SSC-A)/FSC-A plots. Gates in the PI/Annexin V-PB plots were set based on fluorescence minus one controls; Figure S2: GFP intensity on histograms. Intensity of expressed GFP in control samples and different treatments; DMEM 4 h (**a**), DMEM 24 h (**b**), NaF (**c**), OTA (**d**), CHX 4 h (**e**), CHX 24 h (**f**). Four histograms are shown from each example file which represent the four populations defined by propidium iodide (PI) and Annexin V (PI^+^ Annexin V^−^, PI^+^ Annexin V^+^, PI^−^ AnnexinV^+^, PI^−^ Annexin V^−^). We took the highest concentrations from each treatment to be represented in the figure.
