# Supplementary material for: Green Fluorescent Protein-Based Viability Assay in a Multiparametric Configuration
Source: Molecules. 2018 Jun 28;23(7):1575. doi: 10.3390/molecules23071575 (PMC6100089; doi:10.3390/molecules23071575)
Supplement: Supplementary file 1 [file molecules-23-01575-s001.pdf]

Green Fluorescent Protein-Based Viability Assay in a Multiparametric Configuration

Supplementary Materials

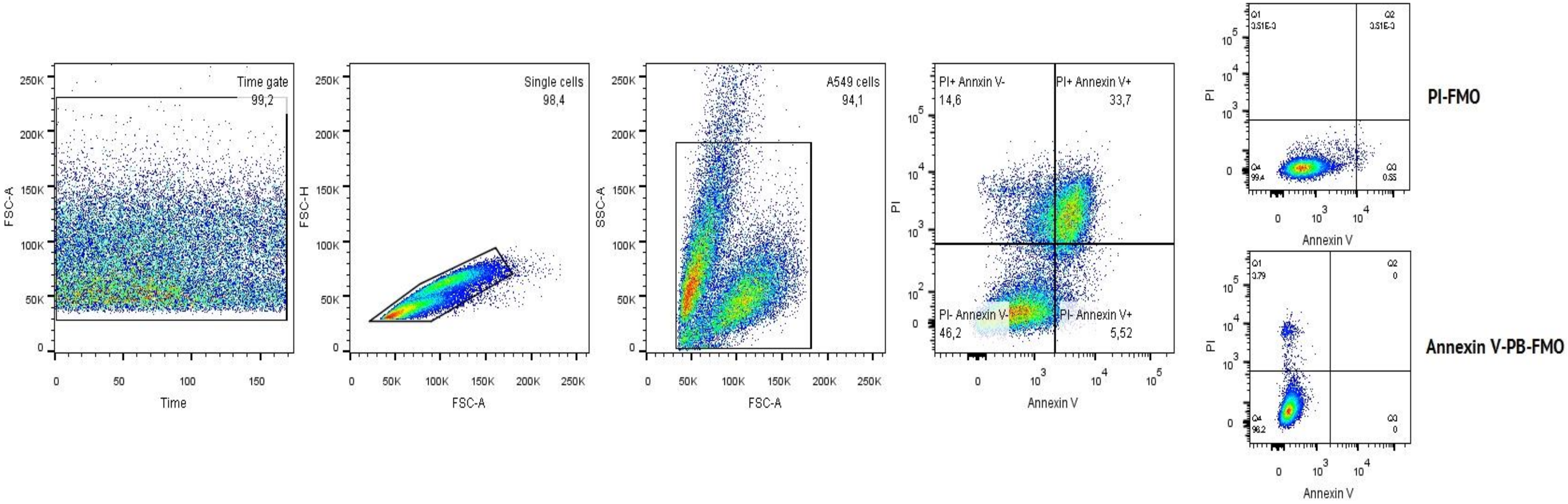

Figure S1. Gating strategy in the flow cytometry experiments. Flow stability was checked by forward scatter area (FSC-A)/Time gate, single cells were discriminated by FSC-Height/FSC-A, A549 cells were determined on side scatter area (SSC-A)/FSC-A plots. Gates in the PI/Annexin V−PB plots were set based on fluorescence minus one controls.

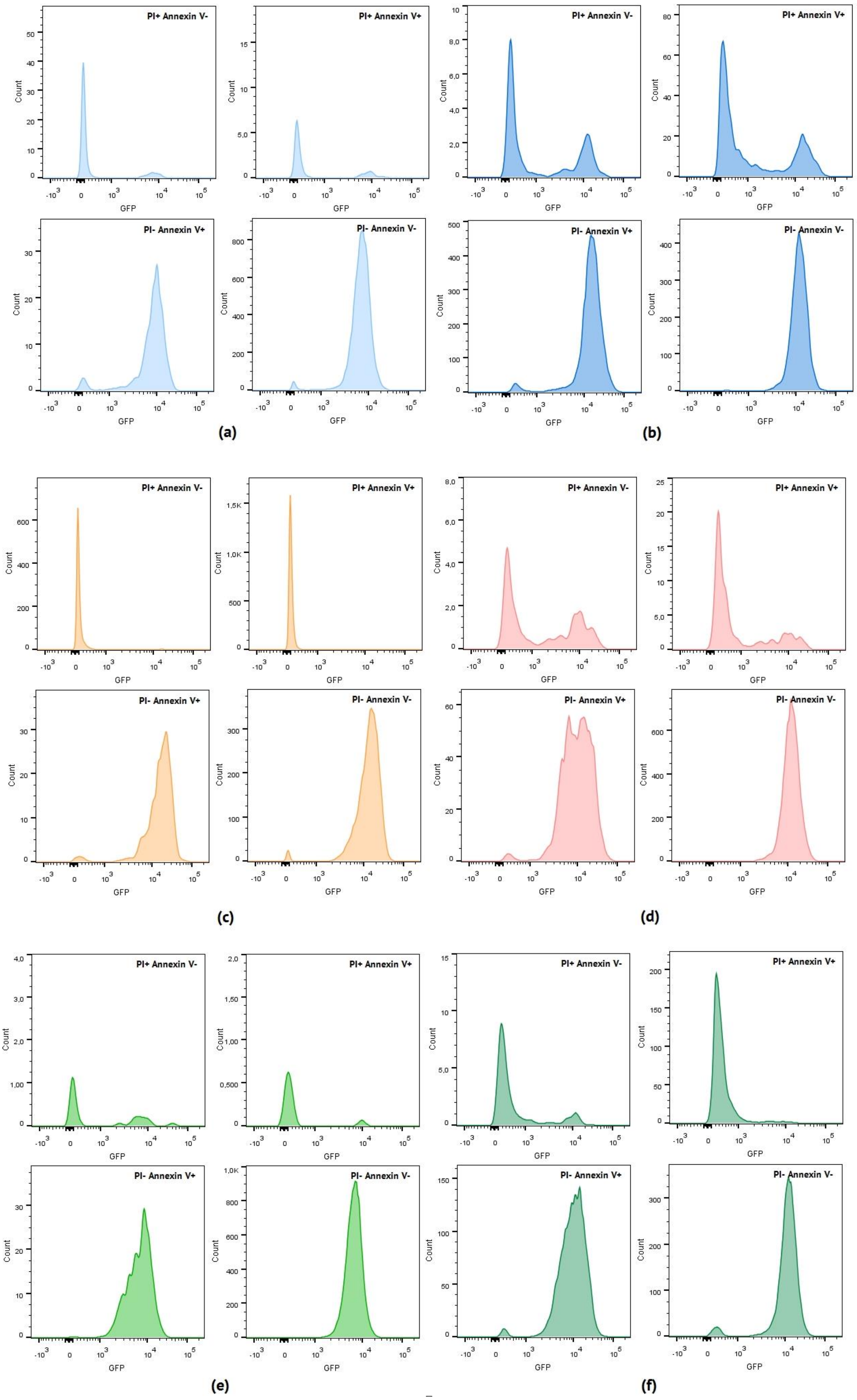
PI+ Annexin V-
PI+ Annexin V+
PI- Annexin V+
PI- Annexin V-
Count
GFP
(a)
(b)
(c)
(d)
(e)
(f)

Figure S2. GFP intensity on histograms. Intensity of expressed GFP in control samples and different treatments; DMEM 4 h (**a**), DMEM 24 h (**b**), NaF (**c**), OTA (**d**), CHX 4 h (**e**), CHX 24 h (**f**). Four histograms are shown from each example file which represent the four populations defined by propidium iodide (PI) and Annexin V (PI^+ Annexin V^-, PI^+ Annexin V^+, PI^- AnnexinV^+, PI^- Annexin V^-). We took the highest concentrations from each treatment to be represented in the figure.
